# Is seeing a specialist nurse associated with positive experiences of care? The role and value of specialist nurses in prostate cancer care

**DOI:** 10.1186/1472-6963-8-65

**Published:** 2008-03-27

**Authors:** Carolyn Tarrant, Paul Sinfield, Shona Agarwal, Richard Baker

**Affiliations:** 1 Department of Health Sciences, University of Leicester, 2nd Floor, Adrian Building, Leicester, LE1 7RH, UK; 2 Department of Health Sciences, University of Leicester, 22-28 Princess Road West, Leicester, LE1 6TP, UK

## Abstract

**Background:**

Specialist nurses may play an important role in helping to improve the experiences of patients with prostate cancer, however there is concern that the specialist nurse role is under threat in the UK due to financial pressures in the NHS. This study explored the role and value of specialist nurses in prostate cancer care via a survey and patient interviews.

**Methods:**

This paper reports findings from two studies. A survey of patients from three hospitals across the UK (289/481, 60%), investigated whether patients who saw a specialist nurse had different experiences of information provision and involvement in decision-making, to those who did not. Qualitative interviews were also carried out with 35 men recently tested or treated for prostate cancer, recruited from two hospitals in the UK. Interviews explored patients' views on the role and value of the specialist nurse.

**Results:**

Survey findings indicated that patients who saw a specialist nurse had more positive experiences of receiving written information about tests and treatment, and about sources of advice and support, and were more likely to say they made the treatment decision themselves. In interviews, patients described specialist nurse input in their care in terms of providing information and support immediately post-diagnosis, as well as being involved in ongoing care. Two key aspects of the specialist nurse role were seen as unique: their availability to the patient, and their ability to liaise between the patient and the medical system.

**Conclusion:**

This study indicates the unique role that specialist nurses play in the experience of patients with prostate cancer, and highlights the importance of maintaining specialist nurse roles in prostate cancer care.

## Background

The care that cancer patients in the UK receive has been under scrutiny, and patients with prostate cancer have been found to report less positive experiences of care than patients with other types of cancer. Patients with prostate cancer are less likely to have the opportunity to discuss side effects of treatment, to understand how well their treatment has gone, or to get information about support and self help groups, than patients with other types of cancer [1].

The role of the specialist nurse in prostate cancer care can be varied, but is primarily focused on the provision of information and support to patients [2]. Specialist nurse involvement may help to improve the experiences of patients with prostate cancer, and may be associated with positive outcomes [3-5]. The NICE Improving Outcomes Guidance on urological cancers emphasised the importance of specialist nurses in prostate cancer care [6], however, prostate cancer patients have lower levels of access to specialist nurses than do patients with other types of cancer [1]. Individuals and organizations involved in prostate cancer care have pointed to the availability of specialist nurses as a key priority for government action [7].

Set against this, current financial pressures in the NHS are threatening specialist nurse posts, with specialist nurses expressing concern that they may face losing their jobs, or may have to give up some or all of their time in their specialist role to work as generalist nurses on hospital wards in order to cover staffing shortages [8-10]. The role of specialist nurses in dealing with information, advice and emotional support, may appear to be "less tangible and a relative 'luxury' when compared with ward-based nurses" [11], leading to their roles being reviewed when resources are constrained. However, Richardson [12] has identified that patients with prostate cancer are more likely to report unmet needs if they do not see a specialist nurse.

There is a need for further research into the role and value of specialist nurses in prostate cancer care, to support decisions about the importance of maintaining and increasing the availability of specialist nurses to patients with prostate cancer.

This paper reports findings from two linked studies carried out as part of a larger study to develop a measure of patient experience of prostate cancer care [13]. A questionnaire survey of patients' experiences of prostate cancer care provided an opportunity to investigate whether patients who saw a specialist nurse had more positive experiences of care than those who did not. Qualitative interviews with patients and carers further explored patient perceptions of the role and value of the specialist nurse in prostate cancer care.

## Methods

Two methodological approaches were used: a quantitative patient survey and a qualitative investigation of patients' experiences.

A questionnaire on experiences of prostate cancer care was mailed to a sample of 481 patients who had been tested or treated for prostate cancer during the previous two years at one of three hospitals in different regions of the UK. Patients were randomly selected from clinic lists for several clinics at each hospital, including patients who were undergoing different types of treatment, and were at different stages in their care. There were no age restrictions put on the sample. Clinic staff were asked to check the lists and exclude any patients who had not been diagnosed, or who were not aware of their diagnosis.

The questionnaire was developed as part of the larger study, and was based on themes identified through interviews of patients with prostate cancer and health professionals [13]. The questionnaire included a question about whether the patient had seen a specialist nurse following their diagnosis. It also included a number of questions relating to the provision of information about treatment options, patient involvement in the treatment decision, and the provision of information about sources of advice and support. Univariate analysis (ANOVA) was used to identify whether patients who saw a specialist nurse had different experiences of these issues to patients who did not see a specialist nurse.

In order to further explore the role of the specialist nurse in prostate cancer care, an analysis of patient interviews undertaken as part of the larger study [13] was also carried out. Interviews were with prostate cancer patients from two hospitals in the East Midlands, UK, recruited using a quota sampling frame to ensure that patients at different stages of disease and treatment, and in different age and ethnicity groups, were included. Patients were identified from attendees at Urology Clinics and from hospitals' patient registers. In addition, two cancer charities were asked to contact patients from ethnic minority groups to ensure that both South Asians and Afro-Caribbeans were represented in the sample.

The interviews were semi-structured and aimed to explore patients' experiences over the course of their care, including initial GP visit(s), further testing, diagnosis, treatment, and ongoing monitoring (where relevant). The interviews did not specifically aim to explore the role of the specialist nurse, although the role and value of the specialist nurse emerged as a theme. Interviews were carried out in patients' own homes; in some cases, the patient's wife or partner was present and also participated in the interview. The interviews were audiotaped and transcribed verbatim, then transferred into the software package NUD*IST 6, and analysed using the Framework approach [14]. Analysis was undertaken to specifically explore the role and value of the specialist nurse in patients' experiences of prostate cancer care. All quotes used have been anonymised, and a patient identification number is given in brackets at the end of each quote.

## Results

### Participants

The questionnaire was completed by 289 patients (60%). Of these, 252 (87.2%) had seen a specialist nurse, and 37 (12.8%) had not. The characteristics of survey responders are given in Table 1. Patients who did not see a specialist nurse were more likely to be in the 75+ age group than patients who did see a specialist nurse (*F *= 8.20; *p *< 0.001), but did not differ significantly in terms of ethnicity, health status, treatment type, or time since most recent treatment.

**Table 1:** Characteristics of questionnaire survey responders (n = 289)

		**Saw specialist nurse **number (%)	**Did not see specialist nurse **number (%)	**Total **number (%)
**Age**	up to 54	6 (2.4)	0	6 (2.1)
	55–64	83 (32.9)	7 (18.9)	90 (31.1)
	65–74	121 (48.0)	14 (37.8)	135 (46.7)
	75 or over	29 (11.5)	16 (43.2)	45 (15.6)
**Ethnicity**	White	211 (83.7)	35 (94.9)	246 (85.1)
	South Asian	5 (2.0)	2 (5.4)	7 (2.4)
	African/Caribbean	21 (8.3)	0	21 (7.3)
	Other	1 (0.4)	0	1 (0.3)
**Stage of disease/treatment**	Newly diagnosed (not yet treated)	0	0	0
	Being actively monitored without treatment	38 (15.1)	10 (27.0)	48 (16.6)
	Had curative treatment (e.g. prostatectomy, radiotherapy)	147 (58.3)	14 (37.8)	161 (55.7)
	Having hormone therapy	52 (20.6)	11 (29.7)	63 (21.8)

Qualitative interviews were carried out with 35 patients; in 10 of the interviews the wife/partner was also present. The characteristics of the patients interviewed are given in Table 2.

**Table 2:** Characteristics of interviewed patients (n = 35)

		**Total **number (%)
**Age**	up to 54	5 (14.3)
	55–70	13 (37.1)
	70 or over	17 (48.6)
**Ethnicity**	White	26 (74.3)
	South Asian	4 (11.4)
	African/Caribbean	5 (14.3)
**Stage of disease/treatment**	Newly diagnosed (not yet treated)	3 (8.6)
	Being actively monitored without treatment	7 (20.0)
	Had curative treatment (e.g. prostatectomy, radiotherapy)	17 (48.6)
	Having hormone therapy	8 (22.9)

### Findings – questionnaire survey

Results from the questionnaire survey were analysed to explore whether patients who saw a specialist nurse had different experiences of care to patients who did not see a specialist nurse. Table 3 shows the odds ratios for questions on patient experience of care, comparing patients who did see a specialist nurse with those who did not, adjusted to take patient age into account. An odds ratio greater than one indicates that a positive response to the question was more likely in the first group (who did see a specialist nurse) than in the second group (who did not see a specialist nurse).

**Table 3:** Odds ratios for questions on patient experience of care: comparison of responses from patients who did, and did not, see a specialist nurse

**Question**	**Saw specialist nurse **Frequency of positive responses/total responses (%)	**Did not see specialist nurse **Frequency of positive responses/total responses (%)	**Age-adjusted odds ratio **Odds ratio, (95% confidence interval) *p value*
1. Given enough written or printed information about the test results	175/225 (77.8)	14/32 (43.8)	4.58 (2.01; 10.43) *p < 0.001*
2. Given enough written or printed information about active treatment	163/195 (83.6)	14/25 (56.0)	3.73 (1.46; 9.56) *p = 0.01*
3. Given enough written or printed information about watchful waiting/active monitoring	127/160 (79.4)	8/22 (36.4)	6.69 (2.45; 18.25) *p < 0.001*
4. Doctor or nurse clearly explained what treatment options would involve	210/239 (87.9)	22/36 (61.1)	3.51 (1.54; 8.01) *p = 0.003*
5. Doctor or nurse discussed clearly the possible side effects or consequences of treatment options	195/239 (81.6)	26/36 (72.2)	1.47 (0.63; 3.45) *p = 0.37*
6. Doctor or nurse gave an explanation of why the other treatment options were not suitable	136/202 (67.3)	15/30 (50)	2.05 (0.92; 4.60) *p = 0.08*
7. Doctor or nurse offered written or printed information about the treatment options	170/250 (68.0)	11/37 (29.7)	3.90 (1.76; 8.63) *p = 0.001*
8. Doctor or nurse offered written or printed information about the side effects or consequences of the treatment options	158/252 (62.7)	10/37 (27.0)	3.81 (1.71; 8.49) *p = 0.001*
9. Patient made decision about which type of treatment to have (alone or in partnership with a health professional)	157/251 (62.5)	11/37 (29.7)	2.69 (1.18; 6.12) *p = 0.02*
10. Doctor or nurse involved patient as much as wanted in the decision about which treatment to have	192/237 (81.0)	23/34 (67.6)	1.69 (0.73; 3.88) *p = 0.22*
11. After the treatment decision had been made, doctor or nurse told patient they could discuss their treatment decision again	152/235 (64.7)	10/34 (29.4)	3.78 (1.68; 8.53) *p = 0.001*
12. Doctor or nurse told patient that they could change their mind about which treatment to have	132/230 (57.4)	6/33 (18.2)	4.71 (1.82; 12.22) *p = 0.001*
13. Doctor or nurse gave patient enough information about sources of help (e.g. support group/charities)	226/252 (89.7)	16/37 (43.2)	9.36 (4.11; 21.34) *p < 0.001*

Patients who saw a specialist nurse were significantly more likely to say that they were given enough written or printed information about their test results and treatment options (Table 3, questions 1, 2, 3, 7 and 8). They were also more likely to feel that their treatment options were clearly explained (Table 3, question 4). Patients who saw a specialist nurse were much more likely to report that they had been given enough information about sources of help (Table 3, question 13). There were no significant differences in terms of whether side effects of treatment were clearly discussed, or whether the doctor or nurse discussed with them why other treatment options were not suitable (Table 3, questions 5 and 6).

Patients who saw a specialist nurse were more likely to say that they made the treatment decision themselves (Table 3, question 9), although there was no significant difference between the groups in the extent to which they felt involved in the treatment decision. Patients who saw a specialist nurse were more likely to have been told that they could discuss the treatment decision again, and could change their mind about treatment (Table 3, questions 11 and 12).

### Findings – qualitative interviews

The analysis of the qualitative interviews explored the role of the specialist nurse in prostate cancer care, with the aim of understanding the differences in experiences of patients who did and did not see a specialist nurse, and the perceived value of the specialist nurse role.

#### Role of the specialist nurse in patients' experiences of prostate cancer care

Most patients first saw the specialist nurse after being given their diagnosis by a consultant. At this stage patients often had to contemplate their diagnosis, consider a range of treatment options, and make a treatment decision. Here the role of the specialist nurse involved providing time for the patient to talk about the diagnosis and ask questions, and providing information about the diagnosis, treatment options, and support services.

[Consultant] said to go with the nurse and she'll explain everything to me, which she did, and made a good job of it ... I was with her about 20 minutes, half an hour...to have things explained and you had the opportunity to ask questions (38)

She was able to talk about the support services that were available in the event of having different options (43)

As well as playing an important role for patients immediately post-diagnosis, specialist nurses provided ongoing support for many patients during the course of treatment and follow-up, through their availability for consultation either by telephone or in person, and in some cases, through arranging patient support groups:

I will go back and be checked from time to time and honestly, my nurse specialist is always there and I will require that service for some time to come (49)

The oncology nurse does run once a month in one of the local pubs in the town a sort of a prostate get together and has done for three or four years probably (15)

#### Unique features of the specialist nurse role

Analysis of patient interviews highlighted the unique nature of the specialist nurse role, and the value of this role to patients. Two key features of the specialist nurse role distinguished it from the roles of other health professionals involved in prostate cancer care. These features were: the availability of the specialist nurse to the patient, and the ability of the specialist nurse to liaise between the medical system and the patient.

##### The availability of the specialist nurse to the patient

Patients described the availability of the specialist nurse firstly in terms of the amount of time the specialist nurse was able to spend with them, and secondly in terms of the specialist nurse's availability for contact throughout their care.

Firstly, patients felt that the specialist nurse was able to spend as much time with them as was needed, and that their time with the nurse was not constrained. This was in direct contrast with the consultant, who was seen as having a limited consultation time. Having this time to talk things over was particularly important for patients after being given their diagnosis. The fact that specialist nurses were available for as much time as the patient needed was highly valued.

I had two, possibly, at least two meetings of hour and a half, two hours ...discussing in detail all the possibilities, all the options, my fears ...She did say herself, take as long as you want, you know, I haven't booked you down for a specific period of time and the first few meetings did take an hour and a half, two hours. Because I had so much to discuss with her (47)

Patients who did not see a specialist nurse after getting their diagnosis highlighted the lack of unconstrained time to talk things over, which had a negative emotional impact on them.

So there I am ... fairly confirmed I would think at that stage that I'm going to need cancer treatment, but no-one really to turn to. That was the thing, that in the whole experience of this, that was the worst moment. I needed somebody ...you know, in a ten minute appointment [consultant]'d really stretched his appointment time I'm sure to give me the benefit of his knowledge ... But that's what I felt I needed, someone to talk to, talk it through (14)

The timing of the consultation with the specialist nurse was important: one patient described seeing the specialist nurse immediately after being given the diagnosis, and felt that this was too soon as he was still in shock following the diagnosis.

No you can't absorb it and that's in a way was one little criticism of [specialist] nurse ... 'you've got prostate cancer' ... and she carts you off into a tiny little cubicle of a room ...and I don't know what the hell she said because ...that was too soon ...I was in a, in a state of numbness anyway at that point and so I don't really know what it was she was trying to achieve (19)

In contrast, one patient described how the specialist nurse had been sensitive to his shock and given him time to come to terms with the diagnosis.

She said to me 'well whatever you are told today you're not gonna take it all in' so they gave me a booklet on prostate cancer and treatments, what is involved and whatever and I was told to go away and read it, and then if I'd got any questions or you know things like that... (54)

A second key aspect of availability that patients valued was the possibility of contact with the specialist nurse for advice and support throughout their care.

Patients were often given the specialist nurse's phone number as a point of contact if they had any concerns or questions. This meant that contact with the specialist nurse was easy, and could be patient-initiated, so patients could have access to support or information as and when they needed it.

It worried me to death ...my mind were in like a whirl. I felt, I had to ring... [specialist nurse], 'cause I ask, I was asking myself questions I couldn't answer, you know 'Why this? And why that?'...after I spoke to her I felt a lot better ...Oh I can ring [specialist nurse] up any time I want to (27)

Just having the contact number was seen as a source of reassurance, whether or not the patient actually needed to use it.

[You can] contact her any time you want to...That's important that you can do that. What does it do, give you sort of reassurance that if you've got a problem or a concern that you can ring, that you know who to ring (38)

Patients who did not have access to this relatively quick and responsive source of support and information had to wait until they had an opportunity, in a scheduled consultation, to discuss issues of concern.

Interviewer: Would you find [contact number for specialist nurse] useful?

Patient: Yes, I would really because if um, odd times I've passed a bit of blood from the bowel and I could ring up and say, 'is that natural?'... You've sort of, you've got to wait till the next appointment, which is three months apart, and that's if they don't cancel it again or nowt (31)

##### The ability of the specialist nurse to liaise between the medical system and the patient

The second unique aspect of the specialist nurse role was that specialist nurses were seen as being in a position to liaise between the medical system and the patient. This involved firstly providing or restating information about diagnosis and treatment in terms which were clearly understandable to the patient, and secondly, acting as an advocate for the patient to facilitate the care process.

Firstly, patients described specialist nurses as helping them to understand and come to terms with their diagnosis and treatment through translating medical information in order to present it in an understandable way. This involved communicating in a patient-centred way and using non-medical language. Specialist nurses were also seen as more likely to address wider issues than simply the diagnosis and treatment, such as the impact of treatments on patients' lifestyles.

Patient: She did explain what the effects of the treatments are, the hormone therapy and so on...

Wife: She was down to earth, she didn't come up with any, you know so many medical terms...and she came up with a lot of practical things that perhaps the consultant wouldn't think to say... the fact that it's affecting your lifestyle (48)

Secondly, patients described this liaison role in terms of specialist nurses acting on their behalf to short-cut delays in care, to gain more information for them, and even to access particular medical services.

She can fiddle about and bang heads in the administration and get things happening (48)

There is two specialist nurses there, I've got their numbers, I speak to them and if there's anything else they will speak to the consultant and then they'll get back to me (54)

I said 'I want... [test]'...My surgeon said ' [patient name] does not require [test]'...But she got it, she got it through another um, consultant (49)

Where specialist nurses were involved in patient support groups, this helped to facilitate this informal liaison role.

When we go to our [patient support] meetings if I say to the oncology nurse ... 'well I've been a little bit worried because...' so she says 'ok don't worry about it I'll see Mr. so and so in the morning I'll give you a ring' (1)

## Discussion

Results from the questionnaire survey indicate that patients who saw a specialist nurse were more likely to have received written information and clear explanations about their tests and treatment options, and about sources of help and support. Patients who saw a specialist nurse were more likely to say that they had made the treatment decision themselves.

The qualitative findings elaborate on and help to explain these differences. In interviews, patients described the contribution of the specialist nurse to their experiences of care immediately post-diagnosis, as well as over the longer term of their treatment and monitoring for prostate cancer. The specialist nurse was primarily seen as providing patients with time to talk and reflect on the diagnosis, providing advice, information and support (including information which could support the patient in making a treatment decision), and in some cases helping to facilitate the course of the patient's care. When patients did not see a specialist nurse, they experienced gaps in their care, in particular, a lack of time to talk things over post-diagnosis, and a lack of immediate access to advice and support over the course of their care.

Importantly, patients' accounts highlight the unique features of the specialist nurse role, which make it possible for specialist nurses to contribute to patients' positive experiences of care. Firstly, patients described the availability of the specialist nurse, in terms of the amount of time the specialist nurse was able to spend with them in contrast with other medical staff such as consultants, and the possibility of patient-initiated contact with the specialist nurse. This concurs with Boxhall and Dougherty's study [15] in which patients valued the extra time available to them with specialist nurses compared to doctors. The second unique aspect of the specialist nurse role was that specialist nurses were seen as being in a position to liaise between the medical system and the patient. This included providing or restating information about diagnosis and treatment in terms which were clearly understandable to the patient, and acting as an advocate for the patient to facilitate the care process. These two key aspects of care have been advocated as important to the specialist nurse role [16], and this study indicates that these aspects of the role are recognised and valued by patients

The unique nature of the specialist nurse role, with their level of availability to the patient and their position at the interface between the patient and the health system, was seen as enabling specialist nurses to address specific patient needs. Some of these needs could not be met by professionals in different roles, as other roles do not share the unique characteristics of the specialist nurse role (for example, consultants are not able to offer patients their time for unlimited periods). Taken together, the findings of the questionnaire survey and the qualitative interviews suggest that specialist nurses make a unique and valuable contribution to patient experience of prostate cancer care.

There are several limitations to the work reported here which should be noted. Firstly, the questionnaire survey was not a randomised controlled study of an intervention, and there is a risk of selection bias. It is possible that patients who did and did not see a specialist nurse differed on factors which were not measured as part of the study. For example, patients who did not see a specialist nurse may have felt less need for nurse input, or may have had more or less advanced disease. This may have had an impact on the study findings, given that only 12.8% of participants had not seen a specialist nurse. However it is notable that the groups did not differ in terms of treatment type or health status. Those who did not see a specialist nurse were older than those who did, and it is possible that some of the participants who did not see a specialist nurse may have been given their diagnosis prior to the widespread input of specialist nurses in care. The survey involved a relatively small number of patients in three hospitals, and responders to the survey were predominantly White British. In addition there were considerable numbers of missing responses on some questions. Although the response rate to the survey was relatively high, the 40% of invited patients who did not respond to the survey may differ systematically to those who did respond, for example, they may be older, or have more advanced disease. Hence the generalisability of the survey results may be limited. The interviews reported here did not systematically explore the role of the specialist nurse; rather this was an issue raised spontaneously by patients, and the analysis is limited to the issues raised by patients. Also, the sampling frame for interviews did not aim specifically to sample those who did and did not see a specialist nurse. The studies do not make a distinction between different types of specialist nurse (e.g. urology specialist nurse and prostate cancer specialist nurse). Nevertheless, the quantitative and qualitative components of the study present complementary findings that together demonstrate the benefits reported by patients of specialist nurses.

## Conclusion

In conclusion, this study indicates that specialist nurses play an important and unique role in prostate cancer care, and have a positive impact on patient experience. It is essential that specialist nurses are supported in their unique role, and that their input is not threatened by financial and organisational pressures.

## Competing interests

The author(s) declare that they have no competing interests.

## Authors' contributions

The authors contributed to the article as follows: CT, PS and RB designed the study. CT, PS and SA collected the data. CT analysed the data with input from PS, SA and RB. CT drew up the draft manuscript and PS, SA and RB contributed to producing the final version.

## Pre-publication history

The pre-publication history for this paper can be accessed here:


